# Bridging knowledge and practice: Antimicrobial Stewardship by community pharmacists in New Zealand

**DOI:** 10.1017/ash.2026.10334

**Published:** 2026-03-27

**Authors:** Vachrisa Stevenson, Hasan Izzat Abdel Rahman, Mudassir Anwar

**Affiliations:** School of Pharmacy, Dunedin Campus: University of Otago, New Zealand

## Abstract

**Objective:**

This study assessed knowledge, perceptions, practices, and barriers regarding Antimicrobial Stewardship (AMS) among community pharmacists in New Zealand.

**Design:**

A cross-sectional study using a self-administered online questionnaire distributed nationwide to community pharmacists.

**Methods:**

A structured questionnaire was distributed via email to 3,226 pharmacists between January and March 2025, assessing demographics, knowledge of antimicrobial resistance (AMR) and antibiotic use, perceptions of AMS, current practices, and barriers. A total of 325 responses underwent quantitative analysis using Mann–Whitney U and Kruskal–Wallis tests (*P* < .05). The Relative Importance Index ranked barriers.

**Results:**

Most respondents (82.8%) perceived community pharmacists as holding a significant role in AMS. While demonstrating strong AMR knowledge (median score 5/7) and positive attitudes (82.7% agreed AMS should be implemented in community pharmacies), practices varied considerably. Patient education on proper antibiotic use was consistent (81.6% always/often), but education on resistance issues was less frequent (40.6% occasionally). Only 27.7% always/often reviewed prescriptions against local guidelines. Primary barriers were lack of time (66.5%), lack of support from higher authorities (64.3%), inadequate staff numbers (61.5%), and limited access to patient records (54.2%). Younger, less experienced pharmacists demonstrated higher knowledge scores (*P* < .05).

**Conclusions:**

Community pharmacists in New Zealand are well-positioned for effective AMS but are hindered by structural and system-level barriers rather than knowledge deficits. Recommendations include developing AMS guidelines tailored to community pharmacists, implementing joint training with prescribers, and establishing annual e-learning refresher modules.

## Introduction

Antimicrobial resistance (AMR) has emerged as one of the most pressing global public health threats, with bacterial AMR directly responsible for 1.27 million deaths globally in 2019.^1^ In response, Antimicrobial Stewardship (AMS) has become a critical strategy to promote responsible antimicrobial use and preserve therapeutic effectiveness for future generations.

The concept of stewardship emerged in the 1990s when AMR levels in hospitals became impossible to ignore.^2,3^ Formal establishment occurred with published guidelines from the Society for Healthcare Epidemiology of America and Infectious Diseases Society of America.^3,4^ These guidelines set the foundation for global adoption of AMS principles, now guided by frameworks such as the World Health Organization’s (WHO) Global Action Plan on AMR.^5^

In New Zealand (NZ), while AMR rates are lower compared to regions such as South-East Asia,^6^ elevated antimicrobial consumption remains concerning. Between 2005 and 2012, annual per capita antimicrobial consumption increased by 43% in community-based patients, with NZ demonstrating one of the highest community antibiotic use rates globally by 2015.^7,8^ Community antibiotic consumption comprises 95% of all antibiotic use in NZ.^9^

The NZ Antimicrobial Resistance Action Plan was released in 2017, followed by AMS guidelines developed by the Best Practice Advocacy Centre (bpacnz).^10,11^ While AMS pharmacists are well-established in hospital settings, with Antimicrobial Stewardship committees operating in NZ District Health Boards,^12^ dedicated outpatient or community-based stewardship pharmacist roles are not formally established in NZ. Unlike hospital settings where AMS pharmacists have defined positions within multidisciplinary teams, community pharmacists currently integrate AMS activities informally within their routine practice without specific role delineation or dedicated time allocation. The role of community pharmacists in AMS efforts remains largely unexplored in local literature.

The NZ community pharmacy landscape comprises approximately 1,100 pharmacies nationwide serving a population of 5.3 million.^13,14^ The community pharmacy sector comprises predominantly independent pharmacies or those owned by small enterprises (57%), franchise-based chains (approximately 30%), and an expanding supermarket-based segment (13%).^15^ Regardless of ownership model, all community pharmacies operate under the same regulatory framework and professional standards, with pharmacists maintaining a consistent scope of practice across settings. However, access to patient health records varies by ownership model and IT infrastructure, with corporate chains often having more integrated systems while independent pharmacies may face greater barriers to electronic health record access.

Community pharmacists, being the most accessible healthcare professionals, are strategically positioned to contribute to AMS through prescription screening, patient education, and collaboration with prescribers.^16^ International studies demonstrate varied knowledge, perceptions, and practices among community pharmacists regarding AMS,^17–25,26–34^ but no research has investigated this specifically in the NZ context. Common barriers identified internationally include a lack of access to patient records, limited collaboration with prescribers, time constraints, and inadequate training.^17–20,24,27–30,34^

This study aimed to fill the information gap by investigating NZ community pharmacists’ knowledge of AMR and appropriate antimicrobial use, their attitudes toward AMS, and their role in combating AMR, current AMS practices, and barriers to effective implementation.

## Methods

### Study design and participants

This quantitative research employed a cross-sectional survey design using a self-administered questionnaire distributed to community pharmacists nationwide between January 30 and March 27, 2025. Inclusion criteria required participants to be NZ-registered community pharmacists.

### Survey design

The questionnaire was developed through international literature review^19,26,27,31,33,34,36,39–41^ and piloted with School of Pharmacy Professional Practice Fellows (n = 2) and three community pharmacists. The final survey consisted of 40 questions across five sections:

1. Demographics (7 items)
2. Knowledge of AMR and antibiotic use (8 items, 3-point Likert scale: always, sometimes, never)
3. Attitudes and perceptions toward AMS and AMR (7 items, 5-point Likert scale: strongly disagree to strongly agree)
4. AMS practices (8 items, 5-point Likert scale: never to always)
5. Barriers to AMS implementation (11 items: 10 structured questions with Yes/Maybe/No responses, plus one open-ended question)

### Ethics approval

The study received ethics approval from the University of Otago Human Ethics Committee (Ref: 24/0700).

### Data collection and recruitment

Following ethics approval, a list of registered pharmacist emails was obtained from the Pharmacy Council. The survey link was distributed via email and social media platforms. Reminder emails were sent fortnightly. An information sheet and poster with QR code were included. Participants could enter a draw to win a $150 Prezzy Card as an incentive.

### Data analysis

Data were analysed using SPSS Version 22. Descriptive analyses (frequencies and percentages) were performed for perceptions and practices. For knowledge assessment, correct responses scored “1” and incorrect responses “0.” Total knowledge scores were calculated, with median scores and quartiles determined.

Inferential statistics (Mann-Whitney U and Kruskal-Wallis tests) compared knowledge, perceptions, and practices across demographic groups (gender, education level, years of experience, age range), accounting for non-normal data distribution. Statistical significance was set at *P* < .05.

The Relative Importance Index (RII) was calculated for barriers using the formula: RII = ΣW / (A × N), where W equals the weighting (1 = No, 2 = Maybe, 3 = Yes), A is the highest weight (3), and N is the total number of participants.

Content analysis was conducted on written responses, with themes identified and frequencies reported.

## Results

### Response rate and demographics

Of 3,226 pharmacists contacted, 412 responded. After excluding incomplete surveys (<90% completion), non-consenting respondents, and non-community pharmacists, 325 responses remained (response rate ∼ 10%). Demographics of the participants are presented in Table 1.

**Table 1.** Demographic characteristics of surveyed community pharmacists

Variable	Category	Frequency (%)
Gender	Male	88 (27.1)
	Female	233 (71.7)
	Prefer not to say	3 (0.9)
	Other	1 (0.3)
Age	20–29	67 (20.6)
	30–39	86 (26.5)
	40–49	60 (18.5)
	50–59	68 (20.9)
	60–69	42 (12.9)
	70 +	1 (0.3)
	Not reported	1 (0.3)
Educational level	Bachelor of pharmacy	205 (63.1)
	Postgraduate certificate in pharmacy	24 (7.4)
	Postgraduate certificate in pharmacist prescribing	2 (0.6)
	Postgraduate diploma in clinical pharmacy	33 (10.2)
	Master of clinical pharmacy	12 (3.7)
	Doctor of philosophy (PHD)	6 (1.8)
	Other	42 (12.9)
	Not reported	1 (0.3)
Practice years	<1 year	1 (0.3)
	1–2 years	33 (10.2)
	3–5 years	35 (10.8)
	6–10 years	42 (12.9)
	>10 years	214 (65.8)

### Knowledge

Almost all respondents (99.4%) had heard of AMR. The median knowledge score was 5 out of a maximum 7 points. The most common score was 6 (35.4% of respondents), with an interquartile range of 1 (Q1 = 5, Q3 = 6), indicating tightly clustered scores.

While most statements showed high correct response rates, notable gaps appeared for “Antibiotics are effective as a first-line treatment for illnesses like the common cold, flu, cough, and sore throat” where only 60.6% correctly answered “never” (Table 2).

**Table 2.** Community pharmacists’ knowledge of AMR and antibiotic use

Statement	Always N (%)	Sometimes N (%)	Never N (%)
Antibiotics are effective as a first-line treatment for illnesses like the common cold, flu, cough, and sore throat.	3 (0.9)	125 (38.5)	197 (60.6)
Antibiotics kill microflora, resulting in secondary infections.	15 (4.6)	278 (85.5)	32 (9.8)
Antibiotics have a prophylactic use.	2 (0.6)	306 (94.2)	17 (5.2)
An antibiotic is a drug that inhibits the growth of bacteria or destroys them.	234 (72.0)	90 (27.7)	1 (0.3)
The misuse of antibiotics leads to a higher risk of antibiotic resistance.	255 (78.5)	70 (21.5)	0
Antibiotic resistance causes infections to spread because standard treatments become less effective.	233 (71.7)	91 (28.0)	1 (0.3)
Antibiotic resistance is when bacteria no longer respond to an antibiotic that should be effective against them.	303 (93.2)	22 (6.8)	0

Significant differences emerged across demographic groups. Younger pharmacists (20–29 years) demonstrated higher knowledge scores compared to older cohorts (40–49, 50–59, 60–69 years). Experience comparisons showed those with 3–5 years were more likely to answer correctly about antibiotics for viral illnesses (*P* = .034). Gender differences appeared for “Antibiotics have a prophylactic use” (*P* = .026), with females more likely to report “always.” Education level significantly affected responses to “Antibiotic resistance is when bacteria no longer respond to an antibiotic” (*P* = .006) (Table 3).

**Table 3.** Comparison of knowledge across demographic groups

Statement	*P* value (Gender comparison)	*P* value (Age comparison)	*P* value (Experience comparison)	*P* value (Education comparison)
Antibiotics are effective as a first-line treatment for illnesses like the common cold, flu, cough, and sore throat.	.560	.315	.034*	.775
Antibiotics kill microflora, resulting in secondary infections.	.906	.380	.400	.881
Antibiotics have a prophylactic use.	.026*	.410	.623	.147
An antibiotic is a drug that inhibits the growth of bacteria or destroys them.	.442	.005*	<.001*	.193
The misuse of antibiotics leads to a higher risk of antibiotic resistance.	.455	.006*	.023*	.213
Antibiotic resistance causes infections to spread because standard treatments become less effective.	.181	.190	.316	.751
Antibiotic resistance is when bacteria no longer respond to an antibiotic that should be effective against them.	.165	.802	.253	.006*

*Statistically significant.

### Perceptions

Strong positive perceptions were evident across most statements. The majority agreed that community pharmacists have a crucial role in reducing antibiotic resistance (82.8% somewhat/strongly agree), over-prescribing contributes to inappropriate use (95.6%), antibiotic resistance is a public health concern (96%), and AMS should be implemented in community pharmacies (82.7%). Most agreed (91.1%) that community pharmacists should receive adequate training on appropriate antimicrobial use (Table 4).

**Table 4.** Community pharmacists’ perceptions of AMR and AMS

Statement	SAN (%)	AN (%)	NN (%)	DN (%)	SDN (%)
Community pharmacists have a crucial role in reducing antibiotic resistance.	112 (34.5)	157 (48.3)	(7.7)	20 (6.2)	11 (3.4)
The over-prescribing of antibiotics contributes to the inappropriate use of antibiotics.	214 (65.8)	97 (29.8)	3 (0.9)	3 (0.9)	8 (2.5)
Antibiotic resistance is a concern for public health.	279 (85.8)	33 (10.2)	2 (0.6)	2 (0.6)	9 (2.8)
Antimicrobial Stewardship (AMS) should be implemented in community pharmacies.	122 (37.5)	147 (45.2)	37 (11.4)	14 (4.3)	5 (1.5)
Community pharmacists should receive adequate training on the appropriate use of antimicrobials.	203 (62.5)	93 (28.6)	15 (4.6)	8 (2.5)	6 (1.8)
I believe that only prescribing physicians need to understand AMS.	32 (9.8)	17 (5.2)	15 (4.6)	89 (27.4)	172 (52.9)
GPs are not open to receiving interventions (from pharmacists) to change the antibiotic they prescribed.	54 (16.6)	144 (44.3)	56 (17.2)	53 (16.3)	18 (5.5)

SA, strongly agree; A, agree; N, neutral; DA, disagree.

Few respondents (15%) believed only physicians need to understand AMS. However, 60.9% agreed that GPs are not open to receiving interventions from pharmacists. Significant variations across age groups (*P* < .001), experience (*P* = .003), and education (*P* = .008) indicated that older pharmacists, those with over 10 years experience, and those with postgraduate diplomas were more likely to disagree with this perception (Table 5).

**Table 5.** Comparison of perceptions across demographic groups

Statement	*P* value (Gender comparison)	*P* value (Age comparison)	*P* value (Experience comparison)	*P* value (Education comparison)
Community pharmacists have a crucial role in reducing antibiotic resistance.	.533	.289	.299	.390
The over-prescribing of antibiotics contributes to the inappropriate use of antibiotics.	.930	.908	.185	.503
Antibiotic resistance is a concern for public health.	.147	.413	.665	.186
Antimicrobial Stewardship (AMS) should be implemented in community pharmacies.	.481	.166	.087	.085
Community pharmacists should receive adequate training on the appropriate use of antimicrobials.	.788	.080	.944	.118
I believe that only prescribing physicians need to understand AMS.	.198	.484	.320	.193
GPs are not open to receiving interventions (from pharmacists) to change the antibiotic they prescribed.	.751	<.001*	.003*	.008*

*Statistically significant.

### Practices

Patient education practices were generally strong. Most respondents (81.6%) always/often educated patients about proper antibiotic use, and 69.2% always advised finishing the full course. However, education on resistance issues was less consistent, with only 38.8% always/often doing so and 40.6% occasionally. Prescription review practices varied considerably. While 75.3% always/often sought additional clinical information before dispensing, only 44.9% always/often checked indications, and merely 27.7% always/often reviewed prescriptions against local guidelines. Before dispensing, 47.4% always/often checked past antibiotic prescribing frequency (Table 6).

**Table 6.** Community pharmacists’ practices surrounding AMS

Statement	Always N (%)	Often N (%)	Occasionally N (%)	Rarely N (%)	Never N (%)
I educate patients about proper use of antibiotics.	101 (31.1)	164 (50.5)	54 (16.6)	5 (1.5)	1 (.3)
I educate patients on issues related to resistance.	24 (7.4)	102 (31.4)	132 (40.6)	58 (17.8)	9 (2.8)
I advise patients to finish the full course of their prescribed antibiotics.	225 (69.2)	78 (24.0)	17 (5.2)	2 (.6)	3 (.9)
I check the indication as to why a patient is being prescribed antibiotics.	36 (11.1)	110 (33.8)	124 (38.2)	50 (15.4)	5 (1.5)
Before dispensing an antibiotic, I check how often a patient has been prescribed antibiotics in the past.	39 (12.0)	115 (35.4)	106 (32.6)	53 (16.3)	11 (3.4)
I review the antimicrobial prescription based on local guidelines before dispensing.	26 (8.0)	64 (19.7)	85 (26.2)	108 (33.2)	41 (12.6)
I reach out to prescribers if I have concerns about the appropriateness of an antibiotic prescription.	96 (29.5)	72 (22.2)	91 (28.0)	59 (18.2)	7 (2.2)
Before dispensing an antibiotic, I seek additional clinical information, such as adverse drug reactions, interactions, and allergies, to ensure safe and appropriate use.	136 (41.8)	109 (33.5)	64 (19.7)	15 (4.6)	0

Significant age-related variation emerged (*P* = .019), with 60–69 year-olds less likely to contact prescribers compared to younger cohorts. Significant differences appeared for patient education on resistance. Younger pharmacists (20–29 years) rarely performed this compared to those aged 50–59 (*P* = .002). Those with over 10 years experience more often educated on resistance compared to those with 1–2 years (*P* < .001). Bachelor of Pharmacy holders were less likely to implement this practice compared to those with other qualifications (*P* = .018) (Table 7).

**Table 7.** Comparison of perceptions across demographic groups

Statement	*P* value (Gender comparison)	*P* value (Age comparison)	*P* value (Experience comparison)	*P* value (Education comparison)
I educate patients about proper use of antibiotics.	.340	.581	.378	.471
I educate patients on issues related to resistance.	.353	.002*	<.001*	.018*
I advise patients to finish the full course of their prescribed antibiotics.	.731	.387	.084	.247
I check the indication as to why a patient is being prescribed antibiotics.	.824	.331	.856	.341
Before dispensing an antibiotic, I check how often a patient has been prescribed antibiotics in the past.	.153	.424	.155	.545
I review the antimicrobial prescription based on local guidelines before dispensing.	.888	.888	.295	.332
I reach out to prescribers if I have concerns about the appropriateness of an antibiotic prescription.	.344	.019*	.613	.465
Before dispensing an antibiotic, I seek additional clinical information, such as adverse drug reactions, interactions,	.192	.771	.507	.341

*Statistically significant.

### Barriers

The Relative Importance Index ranked barriers. The top five barriers were:

1. Lack of time to appropriately implement AMS (RII = 0.866; 66.5% yes)
2. Lack of support and leadership from higher authorities (RII = 0.862; 64.3% yes)
3. Lack of staff numbers (RII = 0.831; 61.5% yes)
4. Lack of access to patient records (RII = 0.795; 54.2% yes)
5. Lack of financial incentives (RII = 0.777; 53.2% yes)

The lowest-ranked barriers were lack of pharmacist interest (RII = 0.533; 48.6% no) and lack of knowledge about AMS (RII = 0.645; 53.8% maybe).

Content analysis of 47 written responses identified key themes. Access to patient information emerged most frequently (14 responses), followed by patient knowledge and expectations (11), limited time and resources (11), and prescriber practices (11). Other themes included collaboration issues (7), guidelines (6), communication barriers (5), external factors (4), staffing issues (3), community versus hospital systems (2), and financial incentives (2).

## Discussion

This study represents the first investigation of community pharmacists’ knowledge, perceptions, practices, and barriers regarding AMS implementation in NZ. The findings reveal that while community pharmacists possess strong knowledge and positive attitudes toward AMS, translation into consistent practice is hindered by structural and system-level barriers.

Community pharmacists demonstrated a solid knowledge foundation, with median scores in the mid-to-high range and a tight interquartile range, suggesting a consistent understanding of AMR and appropriate antibiotic use. These findings align with international studies showing generally good knowledge among community pharmacists.^23,24,26,29,33,38^

However, confusion persisted regarding antibiotic effectiveness for conditions like colds, flu, cough, and sore throat, with only 60.6% providing correct responses. Similar results appeared in a Jordanian study where 56.1% correctly identified antibiotics as never treating viral respiratory infections.^33^ This knowledge gap may stem from the ambiguity of sore throat, which can be bacterial (requiring antibiotics) or viral (not requiring antibiotics), particularly when listed alongside purely viral illnesses.

Younger pharmacists consistently achieved higher knowledge scores than older cohorts, likely reflecting more recent education exposure. This pattern appeared across multiple comparisons: 20–29 year-olds versus 50–59 year-olds for antibiotic definition (*P* = .005) and misuse leading to resistance (*P* = .006). Similarly, less experienced pharmacists (1–2 years, 6–10 years) outperformed those with over 10 years experience on several items. These findings suggest the current Bachelor of Pharmacy curriculum adequately addresses AMR and antibiotic use, but highlight the need for continuing education to maintain knowledge throughout pharmacists’ careers.

Strong positive perceptions emerged across the cohort. The overwhelming majority recognized community pharmacists’ crucial role in reducing antibiotic resistance (82.8%), the link between over-prescribing and inappropriate use (95.6%), AMR as a public health concern (96%), and the need for AMS implementation in community pharmacies (82.7%). These findings mirror international research demonstrating community pharmacists’ awareness of their potential AMS contributions.^19,20,24,27–30,32,35–38^ However, a notable concern emerged: 60.9% believed GPs are not open to pharmacist interventions on antibiotic prescriptions. This perception varied significantly across groups, with late-career and middle-aged pharmacists more likely to disagree, possibly reflecting established professional relationships. Similar findings in Australian studies^18–20^ suggest this barrier transcends national boundaries.

The perception of limited GP cooperation may significantly impact practice. If pharmacists anticipate rejection, they may avoid challenging questionable prescriptions, undermining AMS implementation. This barrier appears more attitudinal than structural, suggesting targeted interventions could improve collaboration.

A disconnect emerged between knowledge/perceptions and practices. While patient education on proper use (81.6% always/often) and finishing courses (69.2% always) was strong, education on resistance issues lagged (38.8% always/often, 40.6% occasionally). This contrasts with Jordanian findings where 59.7% always educated on both antibiotic use and AMR.^33^ The NZ pattern suggests education focuses more on adherence than awareness.

Significant variations appeared across demographics. Younger and less experienced pharmacists were less likely to educate on resistance, despite higher knowledge scores. This paradox may reflect lower confidence in newer practitioners. Conversely, Bachelor of Pharmacy holders were less likely to provide resistance education compared to those with other qualifications, possibly indicating stronger AMS emphasis in postgraduate programs.

Prescription review practices showed considerable inconsistency. While 75.3% always/often checked clinical information, only 27.7% always/often reviewed prescriptions against local guidelines. Similar low guideline adherence appears internationally.^22,39^ Time constraints and limited patient record access likely contribute to this gap.

Prescriber communication varied widely (responses spread from always to rarely), with older pharmacists (60–69) less likely to always contact prescribers compared to younger cohorts (*P* = .019). This finding, juxtaposed with younger pharmacists perceiving less GP openness, suggests generational differences in confidence and approach. Australian research^20^ found pharmacists more readily contacted GPs about interactions, allergies, and doses than appropriateness, while Jordanian pharmacists^33^ more frequently questioned antibiotic choice, indicating varying confidence levels across settings.

Structural and system-level barriers, rather than knowledge deficits, emerged as the primary obstacles. Time constraints ranked highest (RII = 0.866), consistent with international findings,^18,28,30,32,34^ though contrasting with some Australian studies.^19,20^ This reflects the time-constrained nature of community pharmacy, where dispensing demands limit AMS engagement. Notably, lack of pharmacist interest ranked lowest (RII = 0.533), confirming motivation exists, but structural limitations prevent action.

Lack of support from higher authorities ranked second (RII = 0.862), highlighting absent guidance, mentorship, and AMS-specific community pharmacy guidelines. This mirrors international findings^32,34^ and emphasizes that effective AMS implementation requires top-down policy support, not just individual pharmacist effort.

Limited patient record access ranked fourth (RII = 0.795) but dominated written responses (14 mentions), reflecting its practical impact despite moderate ranking. Unlike the complete lack of access reported internationally,^18–20,24,27–30,34^ NZ pharmacists have some access, but systems are time-consuming, and information remains limited. This barrier directly connects to inconsistent prescription review practices.

Collaboration issues with prescribers, while not top-ranked, appeared frequently in written comments, aligning with international findings.^18–20,24,27–30^ Notably, written responses highlighted problematic prescriber practices—particularly prescribing antibiotics for viral infections due to patient demands. This theme, linked to patient knowledge and expectations, underscores the need for public education and the unique position of community pharmacists to monitor prescription appropriateness.

Financial incentives ranked fifth (RII = 0.777), though less emphasized than in some international contexts^22,32,39^ where pharmacy profits from antibiotic sales drive over-dispensing. The moderate ranking in NZ suggests financial concerns exist but are not primary drivers.

Knowledge and training deficits ranked lowest (RII = 0.645 and 0.761 respectively), contrasting sharply with findings from Libya^27^ and Qatar^31^ where knowledge gaps were considered major barriers. This confirms NZ community pharmacists are well-educated and confident in their knowledge, with barriers lying elsewhere.

### Implications and recommendations

Three key recommendations emerge:

*Annual e-learning refresher courses:* Given knowledge score differences across age and experience groups, mandatory yearly modules covering current policies, guidelines, and local AMR patterns would maintain knowledge throughout careers. This addresses knowledge decay in experienced pharmacists while keeping all practitioners current as AMS evolves.

*Joint AMS training with prescribers:* To address collaboration barriers and perceived GP resistance, annual interprofessional training sessions could include collaborative case reviews, discussions on prescribing/dispensing practices, and strategies for teamwork. This would strengthen professional relationships between pharmacists and prescribers, potentially increasing pharmacist confidence in interventions and prescriber receptiveness to collaboration.

*Community pharmacy-specific AMS guidelines:* The absence of tailored guidelines creates ambiguity about pharmacists’ AMS roles and may signal low priority. Developing clear, community-pharmacy-specific AMS guidelines would clarify expectations, support prescription review practices, provide legitimacy for pharmacist interventions, and demonstrate leadership support. Implementation requires top-down commitment but would address the second-ranked barrier directly.

Community pharmacists in NZ demonstrated strong knowledge of AMR and appropriate antibiotic use, coupled with positive perceptions of their role in AMS. However, translation into consistent practice is hindered by structural and system-level barriers rather than knowledge deficits. The primary obstacles—time constraints, lack of organizational support, staffing shortages, and limited patient record access—prevent motivated, knowledgeable pharmacists from fully implementing AMS activities.

These findings emphasize that improving AMS in community pharmacy requires addressing systemic issues through policy changes, resource allocation, and interprofessional collaboration. Development of community pharmacy-specific AMS guidelines, joint training programs with prescribers, and continuing education modules could substantially enhance AMS implementation. Community pharmacists are well-positioned and motivated to combat AMR; they require structural support to fulfil this potential.

## References

[1] World Health Organization. Antimicrobial resistance. Geneva: WHO, 2023. https://www.who.int/news-room/fact-sheets/detail/antimicrobial-resistance. Accessed March 15, 2025

[2] McGowan JE , Jr, Gerding DN. Does antibiotic restriction prevent resistance? New Horiz 1996;4:370–376. 8856755

[3] Charani E , Holmes A. Antibiotic Stewardship-twenty years in the making. Antibiotics (Basel) 2019;8:7. 30678365 10.3390/antibiotics8010007 PMC6466570

[4] David MS , Dale NG , Joseph FJ , et al. Society for healthcare epidemiology of America and infectious diseases society of America joint committee on the prevention of antimicrobial resistance: guidelines for the prevention of antimicrobial resistance in hospitals. Clin Infect Dis 1997;25:584–599. 9314444 10.1086/513766

[5] World Health Organization. Global action plan on antimicrobial resistance. Geneva: WHO, 2016. https://www.who.int/publications/i/item/9789241509763. Accessed August 24, 2025

[6] Ministry of Health. Antimicrobial resistance - Ngā moroiti ārai paturopi. Ministry of Health, 2024. https://www.health.govt.nz/strategies-initiatives/programmes-and-initiatives/antimicrobial-resistance. Accessed March 15, 2025

[7] Thomas MG , Smith AJ , Tilyard M. Rising antimicrobial resistance: a strong reason to reduce excessive antimicrobial consumption in New Zealand. N Z Med J 2014;127:72–84. 24929573

[8] Thomas M. New Zealanders consuming lots of antibiotics without benefit - study. Radio New Zealand, 2018. https://www.rnz.co.nz/news/national/364341/new-zealanders-consuming-lots-of-antibiotics-without-benefit-study. Accessed February 20, 2026

[9] Duffy E , Ritchie S , Metcalfe S , Van Bakel B , Thomas MG. Antibacterials dispensed in the community comprise 85%-95% of total human antibacterial consumption. J Clin Pharm Ther 2018;43:59–64. 28833324 10.1111/jcpt.12610

[10] Te Whatu Ora. New Zealand antimicrobial resistance action plan, 2025. https://www.tewhatuora.govt.nz/for-health-professionals/clinical-guidance/diseases-and-conditions/antimicrobial-resistance/new-zealand-antimicrobial-resistance-action-plan. Accessed February 20, 2026

[11] Bpac NZ. Antimicrobial Stewardship: contextualised from NICE guidelines. Dunedin, New Zealand, 2017.

[12] Gardiner S , Pryer J , Duffy E. Survey of Antimicrobial Stewardship practices in public hospitals in New Zealand district health boards. N Z Med J 2017;130:27–41. 28694537

[13] Pharmacy Council of New Zealand. What is pharmacist practice?, 2024. https://pharmacycouncil.org.nz/public/what-is-pharmacist-practice/. Accessed February 20, 2026

[14] Stats NZ. National population estimates at 31 December 2025. https://www.stats.govt.nz/information-releases/national-population-estimates-at-31-december-2025. Accessed February 20, 2026

[15] Ministry of Health. Pharmacy ownership regulation: regulatory impact statement, 2025. https://www.health.govt.nz/system/files/2025-10/RIS-Pharmacy-ownership-regulation.pdf. Accessed February 20, 2026

[16] Williamson DA , Roberts SA , Ritchie SR , Coombs GW , Fraser JD , Heffernan H. Clinical and molecular epidemiology of methicillin-resistant staphylococcus aureus in New Zealand: rapid emergence of sequence type 5 (ST5)-SCCmec-IV as the dominant community-associated MRSA clone. PLoS One 2013;8:e62020. 23637953 10.1371/journal.pone.0062020 PMC3636228

[17] Saha SK , Kong DCM , Thursky K , Mazza D. Divergent and convergent attitudes and views of general practitioners and community pharmacists to collaboratively implement Antimicrobial Stewardship programs in Australia: a nationwide study. Antibiotics 2021;10:47. 33466476 10.3390/antibiotics10010047 PMC7824809

[18] Saha SK , Kong DCM , Thursky K , Mazza D. Antimicrobial Stewardship by Australian community pharmacists: uptake, collaboration, challenges, and needs. J Am Pharm Assoc (2003) 2021;61:158–68.e7 33187894 10.1016/j.japh.2020.10.014

[19] Rizvi T , Thompson A , Williams M , Zaidi STR. Perceptions and current practices of community pharmacists regarding Antimicrobial Stewardship in Tasmania. Int J Clin Pharm 2018;40:1380–1387. 30069668 10.1007/s11096-018-0701-1 PMC6208572

[20] Rizvi T , Thompson A , Williams M , Zaidi STR. Validation and implementation of a national survey to assess Antimicrobial Stewardship awareness, practices and perceptions amongst community pharmacists in Australia. J Glob Antimicrob Resist 2020;21:28–33. 31505297 10.1016/j.jgar.2019.08.025

[21] Al-Jumaili AA , Ahmed KK. A review of antibiotic misuse and bacterial resistance in Iraq. East Mediterr Health J 2024;30:663–670. 39574365 10.26719/2024.30.10.663

[22] Netthong R , Donsamak S , John DN , Kane R , Armani K. Empowering Thai community pharmacists in combating antimicrobial resistance: qualitative insight and sentiment analysis. Explor Res Clin Soc Pharm 2024;16:100535. 39584022 10.1016/j.rcsop.2024.100535 PMC11584595

[23] Waseem H , Ali J , Sarwar F , et al. Assessment of knowledge and attitude trends towards antimicrobial resistance (AMR) among the community members, pharmacists/pharmacy owners and physicians in district Sialkot. Pak Antimicrob Res Infect Control 2019;8:67. 10.1186/s13756-019-0517-3 PMC6482541 31049196

[24] Atif M , Asghar S , Mushtaq I , Malik I. Community pharmacists as antibiotic stewards: a qualitative study exploring the current status of Antibiotic Stewardship program in Bahawalpur. Pak J Infect Public Health 2020;13:118–124. 10.1016/j.jiph.2019.07.003 31548165

[25] Inayat Ur R , Malik MA , Allah B , et al. Knowledge and practice of pharmacists toward Antimicrobial Stewardship in Pakistan. Pharmacy (Basel) 2018;6:116. 30360517 10.3390/pharmacy6040116 PMC6306925

[26] Akande-Sholabi W , Oyesiji E , Adebisi YA. Antimicrobial Stewardship: community pharmacists’ antibiotic dispensing practices, knowledge, and perception regarding antibiotics and antibiotic resistance. J Pharm Health Serv Res 2023;14:383–391.

[27] Al-Shami HA , Abubakar U , Hussein MSE , Hussin HFA , Al-Shami SA. Awareness, practices and perceptions of community pharmacists towards antimicrobial resistance and Antimicrobial Stewardship in Libya: a cross-sectional study. J Pharm Policy Pract 2023;16:46. 36945072 10.1186/s40545-023-00555-y PMC10028782

[28] Essilini A , Pierre A , Bocquier A , et al. Community pharmacists views on their current role and future opportunities for Antibiotic Stewardship: a French qualitative study. JAC Antimicrob Resist 2021;3:dlab129. 34671729 10.1093/jacamr/dlab129 PMC8521646

[29] Durand C , Chappuis A , Douriez E , et al. Perceptions, current practices, and interventions of community pharmacists regarding Antimicrobial Stewardship: a qualitative study. J Am Pharm Assoc 2022;62:1239–48.e1. 10.1016/j.japh.2022.02.003 35305926

[30] Jones LF , Owens R , Sallis A , et al. Qualitative study using interviews and focus groups to explore the current and potential for Antimicrobial Stewardship in community pharmacy informed by the theoretical domains framework. BMJ Open 2018;8:e025101. 10.1136/bmjopen-2018-025101 PMC6318531 30593557

[31] Pawluk S , Black E , El-Awaisi A. Strategies for improving antibiotic use in Qatar: a survey of pharmacists perceptions and experiences. Int J Pharm Pract 2015;23:77–79. 24650133 10.1111/ijpp.12108

[32] Saleh D , Abu-Farha R , Mukattash TL , Barakat M , Alefishat E. Views of community pharmacists on antimicrobial resistance and Antimicrobial Stewardship in Jordan: a qualitative study. Antibiotics 2021;10:384. 33916855 10.3390/antibiotics10040384 PMC8067308

[33] Darwish RM , Baqain GN , Aladwan H , Salamah LM , Madi R , Masri RMA. Knowledge, attitudes, and practices regarding antibiotic use and resistance among community pharmacists: a cross sectional study in Jordan. Int J Clin Pharm 2021;43:1198–1207. 33515133 10.1007/s11096-021-01234-1

[34] Lee Y , Bradley N. Antimicrobial Stewardship practices in a subset of community pharmacies across the United States. Pharmacy 2023;11:26. 36827664 10.3390/pharmacy11010026 PMC9965000

[35] Haseeb A , Essam Elrggal M , Saeed Bawazir M , et al. Knowledge, attitude, and perception of community pharmacists towards Antimicrobial Stewardship in Saudi Arabia: a descriptive cross-sectional study. Saudi Pharm J 2022;30:1659–1664. 36465854 10.1016/j.jsps.2022.09.010 PMC9715943

[36] Khan MU , Hassali MAA , Ahmad A , Elkalmi RM , Zaidi STR , Dhingra S. Perceptions and practices of community pharmacists towards Antimicrobial Stewardship in the State of Selangor. Malaysia PLoS One 2016;11:e0149623. 26901404 10.1371/journal.pone.0149623 PMC4764324

[37] Tonna AP , Weidmann AE , Sneddon J , Stewart D. Views and experiences of community pharmacy team members on Antimicrobial Stewardship activities in Scotland: a qualitative study. Int J Clin Pharm 2020;42:1261–1269. 32803554 10.1007/s11096-020-01042-z PMC7522063

[38] Gillani SW , Shahwan MKS , Szollosi DE. A questionnaire based survey among pharmacy practitioners to evaluate the level of knowledge and confidence towards Antimicrobial Stewardship. Pharm Pract (1886-3655) 2022;20:1–9. 10.18549/PharmPract.2022.4.2757 PMC9891779 36793910

[39] Hafez H , Rakab MS , Elshehaby A I , et al. Pharmacies and use of antibiotics: a cross-sectional study in 19 Arab countries. Antimicrobial Resistance 2024;13:104. 10.1186/s13756-024-01458-6 PMC11412015 39294829

[40] Chaves NJ , Cheng AC , Runnegar N , Kirschner J , Lee T , Buising K. Analysis of knowledge and attitude surveys to identify barriers and enablers of appropriate antimicrobial prescribing in three Australian tertiary hospitals. Intern Med J 2014;44:568–574. 25083531 10.1111/imj.12373

[41] Abubakar U , Tangiisuran B. Knowledge and practices of community pharmacists towards non-prescription dispensing of antibiotics in Northern Nigeria. Int J Clin Pharm 2020;42:756–764. 32270378 10.1007/s11096-020-01019-y

